# Heat transfer characteristics of magnetized hybrid ferrofluid flow over a permeable moving surface with viscous dissipation effect

**DOI:** 10.1016/j.heliyon.2023.e15907

**Published:** 2023-05-03

**Authors:** Sakinah Idris, Anuar Jamaludin, Roslinda Nazar, Ioan Pop

**Affiliations:** a Department of Mathematics, Universiti Pertahanan Nasional Malaysia, 57000, Kuala Lumpur, Malaysia; b Department of Mathematical Sciences, Faculty of Science and Technology, Universiti Kebangsaan Malaysia, 43600, UKM, Bangi, Selangor, Malaysia; c Department of Mathematics, Babeş-Bolyai University, R-400084, Cluj-Napoca, Romania

**Keywords:** Dual solution, Hybrid ferrofluid, Magnetohydrodynamic, Moving surface, Viscous dissipation

## Abstract

Hybrid ferrofluid is a unique heat transfer fluid because it can be magnetically controlled and ideal in various applications. Further exploration to unleash its potential through studying heat transfer and boundary layer flow is crucial, especially in solving the thermal efficiency problem. Hence, this research focuses on the numerical examination of flow behaviour and heat transfer attributes of magnetized hybrid ferrofluid $Fe_{3}O_{4}-CoFe_{2}O_{4}/$water across a permeable moving surface considering the mutual effects of magnetohydrodynamic (MHD), viscous dissipation, and suction/injection. The problem was represented by the Tiwari and Das model with duo magnetic nanoparticle hybridization; magnetite $Fe_{3}O_{4}$ and cobalt ferrite $CoFe_{2}O_{4}$ immersed in water. The governing equations were transformed into ordinary differential equations using appropriate similarity variables and solved with bvp4c MATLAB. A dual solution is obtained, and via stability analysis, the first solution is stable and physically reliable. The significant influence of governing effects on the temperature and velocity profiles, the local skin friction coefficient and the local Nusselt number are analyzed and visually shown. The surge-up value of suction and $CoFe_{2}O_{4}$ ferroparticle volume concentration enhances the local skin friction coefficient and heat transfer rate. Additionally, the magnetic parameter and Eckert number reduced the heat transfer. Using a 1% volume fraction of $Fe_{3}O_{4}$ and $CoFe_{2}O_{4}$; the hybrid ferrofluid's convective heat transfer rate was shown to be superior to mono-ferrofluid and water by enhancing 2.75% and 6.91%, respectively. This present study also suggests implying a greater volume concentration of $CoFe_{2}O_{4}$ and lessening the magnetic intensity to maintain the laminar flow phase.

## Nomenclature

**Roman letters**
B: magnetic field strength
$C_{f}$: skin friction coefficient
$C_{p}$: specific heat at constant pressure ($Jkg^{-1}K^{-1}$)
${\rho C}_{p}$: heat capacitance of the fluid ($JK^{-1}m^{-3}$)
Ec: Eckert number
f(η): dimensionless stream function
k: thermal conductivity of the fluid ($Wm^{-1}K^{-1}$)
*M*: magnetic parameter
$Nu_{x}$: Nusselt number
Pr: Prandtl number
$q_{w}$: surface heat flux ($Wm^{-3}$)
$Re_{x}$: local Reynolds number
*S*: mass flux parameter (suction/injection)
T: fluid temperature (K)
$T_{w}$: surface temperature (K)
$T_{\infty }$: ambient temperature (K)
t: time (s)
U: constant free stream velocity ($ms^{-1}$)
u,v: velocity component in the x and y directions ($ms^{-1}$)
$v_{w}$: the velocity of the wall mass transfer ($ms^{-1}$)
x,y: cartesian coordinates (m)

**Greek symbols**
γ: eigenvalue
η: similarity variable
θ: dimensionless temperature
r: velocity ratio parameter
μ: dynamic viscosity of the fluid ($kgm^{-1}s^{-1}$)
v: kinematic viscosity of the fluid ($m^{2}s^{-1}$)
ρ: the density of the fluid ($kgm^{-3}$)
τ: dimensionless time variable
$\tau_{w}$: wall shear stress ($kgm^{-1}s^{-2}$)
$\varphi_{1}$: nanoparticle volume fraction for ${\text{Fe}}_{3}O_{4}$ (magnetite)
$\varphi_{2}$: nanoparticle volume fraction for $\text{Co}{\text{Fe}}_{2}O_{4}$ (cobalt ferrite)

**subscripts**
f: base fluid
nf: ferrofluid
hnf: hybrid ferrofluid
$s_{1}$: solid component for ${\text{Fe}}_{3}O_{4}$ (magnetite)
$s_{2}$: solid component for $\text{Co}{\text{Fe}}_{2}O_{4}$ (cobalt ferrite)

**superscript**
′: differentiation to η

## 1 Introduction

Previously, various energy-saving strategies and efforts have been undertaken to minimize industrial costs, enhance heat transmission in heat exchangers, minimize heat transfer time, and boost energy effectiveness. An invention of an intelligent fluid termed nanofluid by Choi and Eastman [1] is one feasible way to enhance the heat transfer coefficient. This compliant strategy influences the thermophysical characteristics of the base fluid, which impacts thermal transfer and fluid flow. A nanofluid can be formed by adding a single nanoparticle (metal oxides, metals, or carbon material) into a base fluid (oil, ethylene glycol, water). Nanofluid benefits numerous industries, including the automotive, electronics, solar energy, biomedicine, and oil recovery sectors [[2], [3], [4], [5], [6]]. Recent discoveries on nanofluid research advancements using different nanoparticles [7,8], including thermal radiation [9,10], viscous dissipation impacts [11,12], non-Newtonian fluid [[13], [14], [15]] as well as in converging and diverging channel [16] can be further explored.

Recently, research paved the way for hybrid nanofluids due to favourable findings gained by nanofluids. A hybrid nanofluid is obtained by mixing several nanoparticles in a based fluid. It has aroused the interest of many scholars, which provides precise control of overheat transport in various industries and offers better thermal conductivity compared to nanofluid and conventional fluids due to the synergistic effect. According to Minea [17], the proper nanoparticle composition and nanoparticle volume concentration to create a hybrid nanofluid should be accurately selected in a study to demonstrate a tremendous positive compatible attribute of each other. Meanwhile, Lund et al. [18] reported that the heat transmission rate is achievable when nanoparticle's volume concentration is 5%, even though most experimental investigations suggested a 5-55% volume fraction for a superior base fluid's rate of thermal transmission and heat conductivity. For instance, Hayat and Nadeem [19] discovered that hybrid nanofluid's thermal conductivity was outstanding compared to nanofluid. Recent exploration implying several pairings of nanoparticles to form hybrid nanofluid in various geometrical surfaces and impacts has been investigated by Refs. [[20], [21], [22], [23], [24]].

Initially, thermal science experts paid less attention to magnetic nanofluid (ferrofluid) until they discovered the involvement of magnetic nanoparticles or ferroparticle such as cobalt ferrite $Co{Fe}_{2}O_{4}$, hematite ${Fe}_{2}O_{3}$, and magnetite $Fe_{3}O_{4}$ boost thermal conductivity. The dispersion of one ferroparticle forms a ferrofluid in a non-magnetic base fluid linked by a colloidal connection. According to recent research, heat transfer increase utilizing a ferrofluid in a magnetic field is more favourable than typical non-magnetic nanofluids [25]. In addition, ferrofluids reduce the cost and size of heat transfer device components [26]. Magnetic fields can govern ferrofluids. Hence, it can be a powerful tool for scientists and super ideal for exploring a new phenomenon extensively utilized in biomedical applications, particularly for magnetic resonance imaging (MRI), magnetotherapy, and thermal therapy used to combat cancer and tumours [27]. They were also applied in devices such as inkjet printers and motion sensors. Abadeh et al. [28] inspect the influence of a continuous magnetic intensity of ferrofluid flow in a helically wrapped coil with a steady wall temperature. Other than that, Kocheril and Elias [29] discovered that magnetized ferrofluid carries more heat than aluminium-based nanofluid. Meanwhile, Mehrez et al. [30] report that the heat exchange can be improved by as much as 86% when ferromagnetic nanoparticles are used with a field magnetism. Alternatively, Jamaludin et al. [31] found that the fluid velocity will accelerate with higher ferroparticles volume concentration. Recently, Hamid et al. [32] scrutinized the particle mobility suspensions in magnetized ferrofluid and discovered that magnetized dust particles hindered ferrofluid flow and expanded the frictional wall's skin.

Many academicians that are interested in resolving real-world heat transfer problems have found hybrid ferrofluids to be intriguing. A hybrid ferrofluid is a unique form of hybrid nanofluid; obtained by mixing a scattered range of magnetic or/and non-magnetic nanoparticles in ferrofluids. It offers enhancement in stability, responsiveness, and magnetic performance compared to conventional ferrofluids. The magnetic nanoparticles impart magnetic characteristics to the fluid, while the liquid matrix stabilizes the nanoparticles and prevents their aggregation. Additionally, the peculiar magnetic features of hybrid ferrofluids, with their low toxicity and high capacity for pollutant removal, have led to increased chemical reactivities [33]. The mixture of magnetic components also offers unique features of hybrid ferrofluids, resulting in characteristics and behaviours that may be adjusted to fit specific needs. Hence it can be magnetically controlled, resulting in an ideal candidate for many applications, especially for drug delivery systems. It has great potential to contribute to various sectors, such as healthcare, energy, and materials research. Applications of hybrid ferrofluid include MRI, drug delivery, magnetic recording medium, sensors, separation, and purification. The use of hybrid ferrofluid as seeds for acid mine drainage (AMD) treatment is an example of an actual application that may be implied from their applications [34]. Thus, choosing a hybrid ferrofluid in this study aims to optimize thermal transmission in the flow field. According to Goharkhah et al. [35], applying a permanent magnetic field in a hybrid ferrofluid dramatically increased heat transfer capacities. On the other hand, Kumar et al. [36] discovered that hybrid ferrofluid offers a more significant transfer rate than ferrofluid, while Anuar et al. [37] report that an increment of a magnetic parameter in hybrid ferrofluid raised local Nusselt number and frictional drag. Due to the constantly developing need for hybrid ferrofluid, excellent research has been conducted, as reported by Refs. [[38], [39], [40]].

The heat transfers and fluid flow problems can be studied experimentally and numerically. Nevertheless, constraint in an experimental study is known to be high in resource, risky, and costly. Therefore, experimental studies used one-step and two-step approaches to produce hybrid nanofluids. Due to its simplicity, the two-step procedure is widely used. Ensuring the hybrid nanofluids' stability is challenging, but the one-step process is highly adaptive to producing nanofluids due to homogeneity. However, it is very costly [41]. As an alternative, the Tiwari and Das, and Buongiorno models are two common mathematical nanofluid models often utilized numerically [42,43]. Akbari et al. [44] demonstrated a numerical dissimilarity between both models. They discovered that under all conditions, the outcomes of the single-phase model are closer to the experimental result. Hence, the Tiwari and Das model is implied in this study to numerically examine the impact of different nanoparticle volume fractions.

Developing a fluid model that enhances flow properties has always been the focal point of many scholars. The efforts do not end with presenting the new model's formulation. They further assess the current concept and investigate its application to the proposed model on various surfaces. Blasius [45] pioneered the issue of boundary layer flow through a fixed surface. Subsequently, Sakiadis [46] anticipates the motion of a boundary layer past a movable surface at a steady speed. Afterwards, the hassle of flow propelled by a moving plate has been studied since it has industrial significance, like cable coating, polymer extrusion, and drawing plastic sheets [47]. Several researchers have investigated the case of plate velocities and continuous free streams flowing in the same or opposing directions [[48], [49], [50]]. Recently, Aladdin et al. [51] and Waini et al. [52] studied hybrid nanofluids in permeable moving surfaces. Both studies report an enhancement of heat transfer with an increment of suction.

Numerous researchers have studied the influence of various effects on the enhancement of fluid stream to determine whether the effects positively impact flow separation. The effect of suction, magnetohydrodynamic (MHD), and viscous dissipation are among the relevant factors that must be considered in fluid flow and thermal efficiency problems. Suction is a way of managing boundary layers that aims to minimize energy waste in channels or friction in outer flow. Specifically, it is intended to prevent or slow down the boundary layer separation [53]. The use of suction can be seen in aerodynamic or fluid flow applications such as pipes and ducts. A study by Waini et al. [54] reported that suction increased the heat transfer of a hybrid nanofluid compared to nanofluid while recently, Wahid et al. [55] reported that velocity profiles decline with a reduction in suction parameter. The combo effects between magnetic fields and fluid motion can be described using MHD, mathematically obtained by Maxwell's electromagnetic and Navier-Stokes fluid dynamics equations. It could regulate flow separation and manipulate heat transfer in certain fluids. The inclusion of MHD in the boundary layer and heat transfer problems has received considerable attention due to its critical practical applications in numerous industrial and engineering fields, such as crystal growth, metal casting, optical grafting, metallurgical process, polymer industry, energy generation, aerospace engineering, medical imaging, and nuclear fusion. According to Rostami et al. [56], magnetic field influence can enhance heat transmission rate and friction drag. In addition, Mehrez and Cafsi [57] discovered that raising the nanoparticle's volume proportion in MHD flow increased the heat transfer ratio. Meanwhile, a recent study by Wahid et al. [58] reported that incorporating Eckert number and radiation parameters into the working fluid shows a reduction in heat transfer rate. Both parameters create an increase in the fluid's thermal state, causing the thermal boundary layer to rise.

Viscous dissipation is a phenomenon that occurs when the fluid viscosity converts the kinetic to thermal energy during the movement of fluid particles. It offers various applications, including heat exchangers, lubrication, polymer processing, microfluidics, and cooling systems. These applications can improve efficiency, control temperature, and prevent damage or overheating by leveraging the heat generated by viscous dissipation. Zainal et al. [59] studied $Al_{2}O_{3}-Cu$/water with the effect of MHD and viscous dissipation. They found Eckert number creates an increase in the fluid's thermal state, hence causing the thermal boundary layer to rise.

The numerical methods to solve the governing equations in fluid flow and heat transfer problems include the shooting method, finite element methods, and bvp4c solver. Additionally, a stability analysis of the resulting solutions (dual solution, i.e., non-unique solution) that exist due to the non-linearity of the equations is crucial to prevent any erroneous interpretation of flow. Thus, the bvp4c MATLAB method rose to prominence owing to its effectiveness in generating a non-unique solution regardless of the complexity of problem formulation with good guess values, as stated by Ref. [60]. Extensive research on the analysis of stability for boundary layer flow and heat transfer problems has been conducted by Weidman et al. [61] and very recently [[62], [63], [64], [65]].

To the best of the authors' knowledge, no prior study corroborates the heat transfer for a parallel hybrid ferrofluid stream with the simultaneous influence of MHD, viscous dissipation, and suction/injection over a permeable moving surface. Thus, a numerical study inspired by Ishak et al. [49] and Waini et al. [52] is presented by broadening their knowledge by including new control parameters such as MHD and viscous dissipation effects. We also considered a different type of fluid compared to the previous studies, a hybrid ferrofluid. The Tiwari and Das model and bvp4c MATLAB program were used to solve the problem numerically. The physical quantities such as local skin friction coefficient $Re_{x}^{1/2}C_{f}$, local Nusselt number $Re_{x}^{-1/2}{Nu}_{x}$, velocity profile $f^{\prime }\left(\eta \right)$, and temperature profiles, θ(η) towards the variation of control parameters are provided graphically and in tabular form. For deeper investigation, the presence of two solutions prompts us to conduct a stability analysis. The novelty of this research lies in the unique and complex flow behavior resulting from the mutual effects of the pertinent governing parameter that arises in this study. Additionally, the numerical solution for a new mathematical formulation development with stability analysis offers a new and better application in various sectors, especially in the cooling and heating process. These novel discoveries are expected to aid researchers or industry in boosting the heat transfer rate through experimentation, cost-effectively implying hybrid ferrofluid.

## 2 Problem formulation

**Fig. 1:**
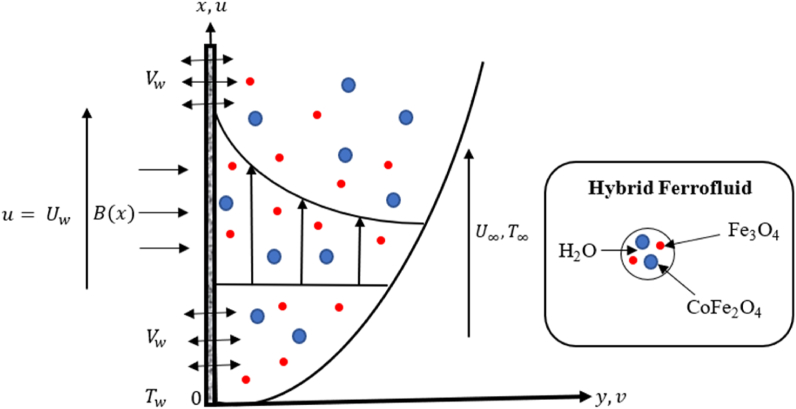
An illustration of physical geometry with a coordinate system.

To develop the model, consider the problem as a two-dimensional steady boundary layer flow and heat transfer of a hybrid ferrofluid consisting of two dissimilar magnetic nanoparticles $Fe_{3}O_{4}-CoFe_{2}O_{4}$ with water base fluid flowing through a half-infinite plate with a uniformly free flow, U. Hybrid ferrofluid is in thermal equilibrium with zero slip conditions between them. Assume the constant velocity $U_{w}$ moving parallel to the free stream $U_{\infty }$ of the two-dimensional moving permeable sheet of a steady laminar flow. The x-axis is aligned with the sheet, whereas the sheet's surface extends vertically normal to the y-axis. Here, the velocity components along the direction of x and y are defined by u and v. $V_{w}\left(x\right)$ indicates the velocity of mass transfer at the sheet's surface. The negative $V_{w}$ implies an injection/blowing, positive $V_{w}$ represent suction whilst $V_{w}=0$ is an impermeable sheet. Meanwhile, $B\left(x\right)=B_{0}{\left(1/2x\right)}^{1/2}$ refers to the uniform magnetic field's strength that was applied orthogonally to the surface being stretched along the positive y-axis, where $B_{0}$ is a constant. Additionally, T is the hybrid ferrofluid temperature whereas $T_{w}$ and $T_{\infty }$ represent constant surface temperature and surrounding temperature, respectively. Thus, the physical geometry to describe the proposed study is given in Fig. 1.

Using typical boundary layer approximations referring to Tiwari and Das model, the improved governing equations is given by [42,49,52,66]

(1) $$\frac{\partial u}{\partial x}+\frac{\partial v}{\partial y}=0\text{,}$$

(2) $$u\frac{\partial u}{\partial x}+v\frac{\partial u}{\partial y}=\frac{\mu_{hnf}}{\rho_{hnf}}\frac{\partial^{2}u}{\partial y^{2}}-\frac{\sigma_{hnf}B^{2}}{\rho_{hnf}}u\text{,}$$

(3) $$u\frac{\partial T}{\partial x}+v\frac{\partial T}{\partial y}=\alpha_{hnf}\frac{\partial^{2}T}{\partial y^{2}}+\frac{\mu_{hnf}}{{\left(\rho C_{p}\right)}_{hnf}}{\left(\frac{\partial u}{\partial y}\right)}^{2}\text{,}$$

subject to the boundary conditions adapting [49]

(4) $$u=U_{w},\;v=V_{w}\left(x\right),\;T=T_{w}\;at\;y=0\text{,}$$

$$u\to U_{\infty },T\to T_{\infty },as\;y\to \infty \text{.}$$

Here, the hybrid ferrofluid's dynamic viscosity is represented by $\mu_{hnf}$, $\rho_{hnf}$ refers to the density of the hybrid ferrofluid, $\sigma_{hnf}$ is the electrical conductivity of the hybrid ferrofluid, the hybrid ferrofluid's thermal diffusivity, $\alpha_{hnf}=k_{hnf}/{\left({\rho C}_{p}\right)}_{hnf}$ where $k_{hnf}$ and ${\left(\rho C_{p}\right)}_{hnf}$ are the thermal conductivity and heat capacitance of the hybrid ferrofluid.

Afzal et al. [67] was a pioneer in declaring a composite velocity $U=U_{w}+U_{\infty }$ to permit the formation of a unique set of equations without separately evaluate the two cases when $U_{w}>U_{\infty }$ and $U_{w}<U_{\infty }$ as determined by [68] and [69].

The subsequent transformation of similarity is initiated to solve Eqs. (1)–(4) by using the following similarity variables as given by [52].

(5) $$\psi =\sqrt{2\nu_{f}xU}f\left(\eta \right),\;\theta \left(\eta \right)=\frac{T-T_{\infty }}{T_{w}-T_{\infty }},\;\eta =y\sqrt{\frac{U}{2x\nu_{f}}}\text{,}$$

where the dimensionless stream function is f, and η is the similarity variable. In addition, the stream function, ψ is defined by u=∂ψ/∂y and v=−∂ψ/∂x, which completely matches Eq. (1). Meanwhile, by using Eq. (5), the following function is obtained by adapting [49,52]

(6) $$u=Uf^{'}\left(\eta \right),\;v=\sqrt{\frac{U\nu_{f}}{2x}}\left(\eta f^{'}\left(\eta \right)-f\left(\eta \right)\right)\text{.}$$

In addition, the existence of similarity solutions for Eqs. (1)–(4) is ensured implying [52,70]

(7) $$V_{w}\left(x\right)=-\sqrt{\frac{U\nu_{f}}{2x}}S\text{.}$$

The dimensionless constant, *S* in Eq. (7), determines the surface transpiration rate where the condition of S<0 and S>0 represent the suction and injection, respectively. Meanwhile, the condition of S=0 indicates an impermeable sheet.

Further, Eqs. (5), (6) are applied to reduced Eqs. (2), (3) to:

(8) $$\frac{\mu_{hnf}}{\mu_{f}}f^{\prime \prime \prime }-\frac{\sigma_{hnf}}{\sigma_{f}}Mf^{\prime }+\frac{\rho_{hnf}}{\rho_{f}}ff\prime \prime =0\text{,}$$

(9) $$\frac{1}{\mathrm{Pr}}\frac{k_{hnf}}{k_{f}}\theta^{\prime \prime }+\frac{{\left(\rho C_{p}\right)}_{hnf}}{{\left(\rho C_{p}\right)}_{f}}f\theta^{\prime }+\frac{\mu_{hnf}}{\mu_{f}}Ec{\left(f^{\prime \prime }\right)}^{2}=0\text{,}$$

where the Eckert number Ec, Prandtl number Pr⁡, and magnetic parameter M are defined as:

(10) $$\mathrm{Pr}=\frac{{\left(\mu C_{p}\right)}_{f}}{k_{f}},\;M=\frac{\sigma_{f}B_{0}^{2}}{\rho_{f}U},\;Ec=\frac{U^{2}}{{\left(C_{p}\right)}_{f}\left(T_{w}-T_{\infty }\right)}\text{.}$$

It should be emphasized that *M* and Ec, as specified in Eq. (10), are the pertinent controlling parameters that arise due to the involvement of MHD and viscous dissipation impact in Eqs. (3), (4), respectively.

Furthermore, using Eqs. (5), (6), the boundary conditions (4) become:

(11) $$f\left(0\right)=S,\;f^{'}\left(0\right)=1-r,\;\theta \left(0\right)=1,\;f^{'}\left(\eta \right)\to r,\;\theta \left(\eta \right)\to 0\;\mathrm{as}\;\eta \to \infty \text{,}$$

where the velocity ratio parameter denoted by r is defined by Cortell [48] and Afzal et al. [67] as:

(12) $$r=\frac{U_{\infty }}{U}\text{.}$$

In Eq. (12), 0<r<1 corresponds to the sheet moving parallel according to the route of the free stream when. Meanwhile, when r<0 and r>1 represent a sheet moving in the opposite direction. We discovered that when an impermeable sheet and M=0, Eqs. (8), (9) are identical to Cortell [48] and Afzal et al. [67]. In contrast, when r=1 and r=0, respectively, it coincide with the traditional Blasius [45] and Sakiadis [46] problems.

The practical concern of physical quantities in this study are the Nusselt number $Nu_{x}$ and skin friction coefficient $C_{f}$ expressed as follows:

(13) $$Nu_{x}=\frac{xq_{w}}{k_{f}\left(T_{w}-T_{\infty }\right)},\;C_{f}=\frac{\tau_{w}}{\rho_{f}U^{2}}\text{,}$$

where the surface's heat flux, $q_{w}$ and the wall shear stress, $\tau_{w}$ is represented by:

(14) $$q_{w}=-k_{hnf}{\left(\frac{\partial T}{\partial y}\right)}_{y=0},\;\tau_{w}=\mu_{hnf}{\left(\frac{\partial u}{\partial y}\right)}_{y=0}\text{.}$$

Upon plugging in Eqs. (5), (6) into Eqs. (13), (14) turned to:

(15) $$\;Re_{x}^{1/2}C_{f}=\frac{1}{\sqrt{2}}\frac{\mu_{hnf}}{\mu_{f}}f^{''}\left(0\right),\;\;Re_{x}^{-1/2}Nu_{x}=-\frac{1}{\sqrt{2}}\frac{k_{hnf}}{k_{f}}\theta^{'}\left(0\right)\text{.}$$

In this case, $Re_{x}^{1/2}C_{f}$ and $Re_{x}^{-1/2}{Nu}_{x}$ in Eq. (15) denoted as a local skin friction coefficient and local Nusselt number, correspondingly. Meanwhile, ${Re}_{x}=Ux/\nu_{f}$ is the local Reynolds number.

It is worth noting that the intended hybrid ferrofluid in the current study is produced by the dissolving 1% of magnetite ${Fe}_{3}O_{4}$ in a base fluid (water). The ${Fe}_{3}O_{4}/$water ferrofluid is then mixed with cobalt ferrite $Co{Fe}_{2}O_{4}$ to create a $Fe_{3}O_{4}-CoFe_{2}O_{4}/$water hybrid ferrofluid. Moreover, the volume fraction of $Co{Fe}_{2}O_{4}$ ferroparticles varies between 0-5%. The base fluid, magnetic and non-magnetic nanoparticles' thermophysical properties, as provided by Anuar et al. [37], are shown in Table 1. In addition, the ferrofluid and hybrid ferrofluid thermophysical properties rely on the work of Devi and Devi [66] and Oztop and Abu Nada [71], where the equations are supplied in Table 2.

**Table 1:** Thermophysical properties of water, magnetic, and non-magnetic nanoparticles [37].

	Physical Properties			
	$C_{p}\left(Jkg^{-1}K^{-1}\right)$	$\rho \left(kgm^{-3}\right)$	$k\left(Wm^{-1}K^{-1}\right)$	$\sigma \left(Sm^{-1}\right)$
Water ($H_{2}O)$	4179	997.1	0.613	0.05
Magnetic nanoparticles				
Magnetite (${Fe}_{3}O_{4})$	670	5180	9.7	$0.74\times 10^{6}$
Cobalt ferrite ($Co{Fe}_{2}O_{4})$	700	4907	3.7	$1.1\times 10^{7}$
Non-magnetic nanoparticle				
Copper (Cu)	385	8933	401	$5.96\times 10^{7}$
Alumina ($Al_{2}O_{3})$	765	3970	40	$1\times 10^{-10}$

**Table 2:** Thermophysical properties of ferrofluid and hybrid ferrofluid (Devi & Devi [66]; Oztop & Abu Nada [71]).

Properties	Ferrofluid	Hybrid Ferrofluid
Viscosity	$\mu_{nf}=\frac{\mu_{f}}{{\left(1-\varphi \right)}^{2.5}}$	$\mu_{hnf}=\frac{\mu_{f}}{{\left(1-\varphi_{1}\right)}^{2.5}{\left(1-\varphi_{2}\right)}^{2.5}}$
Density	$\rho_{nf}=\left(1-\varphi_{1}\right)\rho_{f}+\varphi_{1}\rho_{s1}$	$\rho_{hnf}=\left(1-\varphi_{2}\right)\left[\left(1-\varphi_{1}\right)\rho_{f}+\varphi_{1}\rho_{s1}\right]+\varphi_{2}\rho_{s2}$
Electrical conductivity	$\frac{\sigma_{nf}}{\sigma_{f}}=1+\frac{3\left(\sigma_{s1}/\sigma_{f}-1\right)\varphi_{1}}{\sigma_{s1}/\sigma_{f}+2-\left(\sigma_{s1}/\sigma_{f}-1\right)\varphi_{1}}$	$\frac{\sigma_{hnf}}{\sigma_{f}}=\frac{\sigma_{s2}+2\sigma_{nf}-2\varphi_{2}\left(\sigma_{nf}-\sigma_{s2}\right)}{\sigma_{s2}+2\sigma_{nf}+\varphi_{2}\left(\sigma_{nf}-\sigma_{s2}\right)}$ where, $\frac{\sigma_{nf}}{\sigma_{f}}=\frac{\sigma_{s1}+2\sigma_{f}-2\varphi_{1}\left(\sigma_{f}-\sigma_{s1}\right)}{\sigma_{s1}+2\sigma_{f}+\varphi_{1}\left(\sigma_{f}-\sigma_{s1}\right)}$
Thermal diffusivity	$\alpha_{nf}=\frac{k_{nf}}{{\left({\rho C}_{p}\right)}_{nf}}$	$\alpha_{hnf}=\frac{k_{hnf}}{{\left({\rho C}_{p}\right)}_{hnf}}$
Thermal expansion coefficient	${\left(\rho \beta \right)}_{nf}=\left(1-\varphi_{1}\right){\left(\rho \beta \right)}_{f}+\varphi_{1}{\left(\rho \beta \right)}_{s1}$	${\left(\rho \beta \right)}_{hnf}=\left(1-\varphi_{2}\right)\left[\left(1-\varphi_{1}\right){\left(\rho \beta \right)}_{f}+\varphi_{1}{\left(\rho \beta \right)}_{s1}\right]+\varphi_{2}{\left(\rho \beta \right)}_{s2}$
Heat capacity	${\left({\rho C}_{p}\right)}_{nf}=\left(1-\varphi_{1}\right){\left({\rho C}_{p}\right)}_{f}+\varphi_{1}{\left({\rho C}_{p}\right)}_{s1}\text{,}$	${\left({\rho C}_{p}\right)}_{hnf}=\left(1-\varphi_{2}\right)\left[\left(1-\varphi_{1}\right){\left({\rho C}_{p}\right)}_{f}+\varphi_{1}{\left({\rho C}_{p}\right)}_{s1}\right]+\varphi_{2}{\left({\rho C}_{p}\right)}_{s2}$
Thermal conductivity	$\frac{k_{nf}}{k_{f}}=\frac{k_{s1}+2k_{f}-2\varphi_{1}\left(k_{f}-k_{s1}\right)}{k_{s1}+2k_{f}+\varphi_{1}\left(k_{f}-k_{s1}\right)}$	$\frac{k_{hnf}}{k_{f}}=\frac{k_{s2}+2k_{nf}-2\varphi_{2}\left(k_{nf}-k_{s2}\right)}{k_{s2}+2k_{nf}+\varphi_{2}\left(k_{nf}-k_{s2}\right)}$ where, $\frac{k_{nf}}{k_{f}}=\frac{k_{s1}+2k_{f}-2\varphi_{1}\left(k_{f}-k_{s1}\right)}{k_{s1}+2k_{f}+\varphi_{1}\left(k_{f}-k_{s1}\right)}$

## 3 Stability analysis

According to the current investigation, the numerical outcome confirmed the occurrence of the dual solutions (first and second solutions). Thus, it is compulsory to examine the stability of the dual solutions obtained in Eqs. (8), (9) with boundary conditions (11). Hence, the current problem was revised by maintaining Eq. (1) while Eqs. (2), (3) were deemed unsteady as follows:

(16) $$\frac{\partial u}{\partial t}+u\frac{\partial u}{\partial x}+v\frac{\partial u}{\partial y}=\frac{\mu_{hnf}}{\rho_{hnf}}\frac{\partial^{2}u}{\partial y^{2}}-\frac{\sigma_{hnf}B^{2}}{\rho_{hnf}}u,$$

(17) $$\frac{\partial T}{\partial t}+u\frac{\partial T}{\partial x}+v\frac{\partial T}{\partial y}=\alpha_{hnf}\frac{\partial^{2}T}{\partial y^{2}}+\frac{\mu_{hnf}}{{\left(\rho C_{p}\right)}_{hnf}}{\left(\frac{\partial u}{\partial y}\right)}^{2},$$

where t denotes the time. Next, we adopt the following new similarity variables as established by [52]

(18) $$v=\sqrt{\frac{Uv_{f}}{2x}}\left[\eta \frac{\partial f}{\partial \eta }(\eta ,\tau )-f(\eta ,\tau )+2\tau \frac{\partial f}{\partial \tau }(\eta ,\tau )\right],\;\;u=U\frac{\partial f}{\partial \eta }\left(\eta ,\tau \right),\;\eta =y\sqrt{\frac{U}{2x\nu_{f}}},\;\;\theta \left(\eta ,\tau \right)=\frac{T-T_{\infty }}{T_{w}-T_{\infty }},\;\tau =\frac{Ut}{2x}.$$

Then, substituting Eq. (18) into Eqs. (16), (17), we obtained:

(19) $$\left(\frac{\mu_{hnf}}{\mu_{f}}\right)\frac{\partial^{3}f}{\partial \eta^{3}}-\left(\frac{\sigma_{hnf}}{\sigma_{f}}\right)M\frac{\partial f}{\partial \eta }+\left(\frac{\rho_{hnf}}{\rho_{f}}\right)\left[f\frac{\partial^{2}f}{\partial \eta^{2}}-\frac{\partial^{2}f}{\partial \eta \partial \tau }+2\tau \left(\frac{\partial f}{\partial \eta }\frac{\partial^{2}f}{\partial \eta \partial \tau }-\frac{\partial f}{\partial \tau }\frac{\partial^{2}f}{\partial \eta^{2}}\right)\right]=0\text{,}$$

(20) $$\frac{1}{\mathrm{Pr}}\frac{k_{hnf}}{k_{f}}\frac{\partial^{2}\;\theta }{\partial \eta^{2}}+\frac{\mu_{hnf}}{\mu_{f}}Ec{\left(\frac{\partial^{2}f}{\partial \eta^{2}}\right)}^{2}+\frac{{\left(\rho C_{p}\right)}_{hnf}}{{\left(\rho C_{p}\right)}_{f}}\left[f\frac{\partial \theta }{\partial \eta }-\frac{\partial \theta }{\partial \tau }+2\tau \left(\frac{\partial f}{\partial \eta }\frac{\partial \theta }{\partial \tau }-\frac{\partial f}{\partial \tau }\frac{\partial \theta }{\partial \eta }\right)\right]=0\text{,}$$

and the boundary conditions in Eq. (11) then transform to:

(21) $$f\left(0,\tau \right)-2\tau \frac{\partial f}{\partial \tau }=S,\;\frac{\partial f}{\partial \eta }\left(0,\tau \right)=1-r,\;\theta \left(0,\tau \right)=1,\;\;\frac{\partial f}{\partial \eta }\left(\eta ,\tau \right)\to r,\;\theta \left(\eta ,\tau \right)\to 0,\;as\;\eta \to \infty .$$

To ascertain the solution's steady flow stability and allow the disturbance in Eqs. (19), (20), (21), which compliance to Weidman et al. [61], we employed the following functions

(22) $$f\left(\eta ,\tau \right)=f_{0}\left(\eta \right)+e^{-\gamma \tau }F\left(\eta \right),\;\;\theta \left(\eta ,\tau \right)=\theta_{0}\left(\eta \right)+e^{-\gamma \tau }G\left(\eta \right),$$

where F(η) and G(η) are relatively small, respecting to ${f=f}_{0}\left(\eta \right)$ and $\theta =\theta_{0}\left(\eta \right)$. Meanwhile, γ indicates the eigenvalue used to provide the rate of growth or decay of disturbances to the steady-state similarity solution $f_{0}\left(\eta \right)$ and $\theta_{0}\left(\eta \right)$. Hence by setting up τ=0 in Eq. (22), the initial growth or decay of the solution could be identified. Therefore, the simplified linearized equations are given by

(23) $$\frac{\mu_{hnf}}{\mu_{f}}F^{\prime \prime \prime }-\frac{\sigma_{hnf}}{\sigma_{f}}MF^{\prime }+\frac{\rho_{hnf}}{\rho_{f}}\left(f_{0}F+f_{0}F+\gamma F^{\prime }\right)=0\text{,}$$

(24) 1PrkhnfkfG″+(ρCp)hnf(ρCp)f(f0G'−θ0'F+γG)+2μhnfμfEc(f0″F″)=0,

subject to boundary conditions

(25) $$F\left(0\right)=0,\;F^{'}\left(0\right)=0,\;G\left(0\right)=0,\;\;F^{'}\left(\eta \right)\to 0,\;G\left(\eta \right)\to 0\;\mathrm{as}\;\eta \to \infty .$$

The systems of linearized equations (23), (24), (25) will exhibit an endless number of eigenvalues $\gamma_{1}<\gamma_{2}<\ldots$ where $\gamma_{1}$ is the smallest eigenvalue. In other words, Eqs. (23), (24) are unsolvable using boundary conditions of Eq. (25). Hence, a boundary condition's relaxation at η→∞ are carried out by replacing either $F^{'}\left(\eta \right)\to 0$ to $F^{\prime \prime }\left(0\right)=1$ or $G\left(\eta \right)\to 0$ to $G^{'}\left(0\right)=1$ as reported by Ref. [64]. Here, $F^{'}\left(\infty \right)\to 0$ was chosen to be relaxed and switched to $F^{\prime \prime }\left(0\right)=1$, resulting new boundary conditions as follows

(26) $$F\left(0\right)=0,\;F^{'}\left(0\right)=0,\;F^{''}\left(0\right)=1,\;G\left(0\right)=0,\;\;G\left(\eta \right)\to 0\;\mathrm{as}\;\eta \to \infty .$$

The smallest eigenvalue is attained by solving Eqs. (23), (24) subject to Eq. (26). The negative $\gamma_{1}$ reveals the unstable solution that can cause additional disruption. In the meantime, a positive $\gamma_{1}$ represents a stable solution that can degenerate the disturbance.

## 4 Methodology and validation

The bvp4c MATLAB programming system was used for the mathematical computations for this study endeavor, which were subject to Eqs. (8), (9), as well as the boundary conditions in Eq. (11). An early determination of the principal mesh point and modifications step size is critical for obtaining desired results. Furthermore, a plausible assumption of boundary layer thickness and effective preliminary approximation is required to generate the non-uniqueness solutions. Thus, a suitably finite value of η→∞ and the relative error tolerance on residuals for this problem is particularly set to $\eta =\eta_{\infty }=14$ and $10^{-5}$. Therefore, the boundary value problem should be simplified to a set of ordinary differential equations, which can then be solved. The resulting Eq. (8) is expressed as

(27) $$f=y\left(1\right),\;f^{'}=y^{'}\left(1\right)=y\left(2\right),\;f^{''}=y^{'}\left(2\right)=y\left(3\right),\;f^{'''}=y^{'}\left(3\right)=\frac{1}{\mu_{hnf}/\mu_{f}}\left[\frac{\sigma_{hnf}}{\sigma_{f}}My\left(2\right)-\frac{\rho_{hnf}}{\rho_{f}}y\left(1\right)y\left(3\right)\right],$$

whereas Eq. (9) becomes

(28) $$\theta =y\left(4\right),\;\theta^{'}=y^{'}\left(4\right)=y\left(5\right),\;\theta^{''}=-\frac{\mathrm{Pr}}{k_{hnf}/k_{f}}\left[\frac{{\left(\rho C_{p}\right)}_{hnf}}{{\left(\rho C_{p}\right)}_{f}}y\left(1\right)y\left(5\right)+\frac{\mu_{hnf}}{\mu_{f}}Ec{\left(y\left(3\right)\right)}^{2}\right].$$

Further, the boundary conditions (11) now turn to

(29) $$ya\left(1\right)=S,\;ya\left(2\right)=1-r,\;ya\left(4\right)=1,\;yb\left(2\right)=r,\;yb\left(4\right)=0.$$

Eqs. (27), (28), (29) are then encoded into MATLAB's bvp4c function. Solver syntax sol = bvp4c (@OdeBVP, @OdeBC, solinit, options) includes the function handle @OdeBVP, into which Eqs. (27), (28) are encoded. Afterwards, the boundary conditions in Eq. (29) are encoded into the operation handle @OdeBC. The “solinit” function codes the initial mesh points and the initial solution approximation at the mesh points. Moreover, the “options” function is an optional integration argument. The solver will execute, and numerical solutions and graphs will be displayed as output.

Several validations to correlate the literature's results were done to ensure that the employing bvp4c in MATLAB is accurate and capable of overcoming the current problem. The acquired data to examine the effects of physical characteristics are subjected to nanoparticles volume concentrations $\varphi_{1}$ and $\varphi_{2}\text{,}$ velocity ratio parameter r, Eckert number Ec, magnetic parameter M, and injection/suction parameter S. Note that, $\varphi_{1}$ and $\varphi_{2}$ represent the volume concentration of magnetite ${Fe}_{3}O_{4}$ and cobalt ferrite $Co{Fe}_{2}O_{4}$ ferroparticles, respectively. To achieve the numerical findings; $\varphi_{1}$ = 0.01 and Pr=6.2 (since the water was used as base fluid) is maintained throughout conducting this study, while other parameters are varied within the following range; $0\leq \varphi_{2}\leq 0.05$, −0.1≤S≤0.2, 0≤M≤0.03, 0≤Ec≤0.05, and 0≤r<2. Hence, Table 3, Table 4 compare the present work with numerical values obtained from Cortell [48], Ishak et al. [49], and Waini et al. [52]. The provided results were in excellent agreement and are extremely compatible.

**Table 3:** Comparative values of $f^{\prime \prime }\left(0\right)$ for common liquid $(\varphi_{1}=\varphi_{2}=0$) when S=0 and r=1.

Cortell [48]	Ishak et al. [49]	Waini et al. [52]	Present
0.469602	0.469601	0.469600	0.469600

**Table 4:** Comparative values of $Re_{x}^{1/2}C_{f}$ and $Re_{x}^{-1/2}{Nu}_{x}$ for various value $\varphi_{2}$ for Cu/water nanofluids $(\varphi_{1}=0$) and $Cu-Al_{2}O_{3}$/water hybrid nanofluid $(\varphi_{1}=0.1$) when S=0,r=1 and Pr=6.2.

	Cu/ water				$Cu-Al_{2}O_{3}/$ water			
	$Re_{x}^{1/2}C_{f}$		$Re_{x}^{-1/2}{Nu}_{x}$		$Re_{x}^{1/2}C_{f}$		$Re_{x}^{-1/2}{Nu}_{x}$	
$\varphi_{2}$	Waini et al. [52]	Present	Waini et al. [52]	Present	Waini et al. [52]	Present	Waini et al. [52]	Present
0.1	0.498390	0.498390	0.657797	0.657797	0.609544	0.609544	0.775147	0.775147
0.01	0.349380	0.349380	0.637362	0.637362	0.449759	0.449759	0.755153	0.755153
0.05	0.418605	0.418605	0.704281	0.704281	0.523678	0.523678	0.826686	0.826686

## 5 Results and discussion

**Fig. 2:**
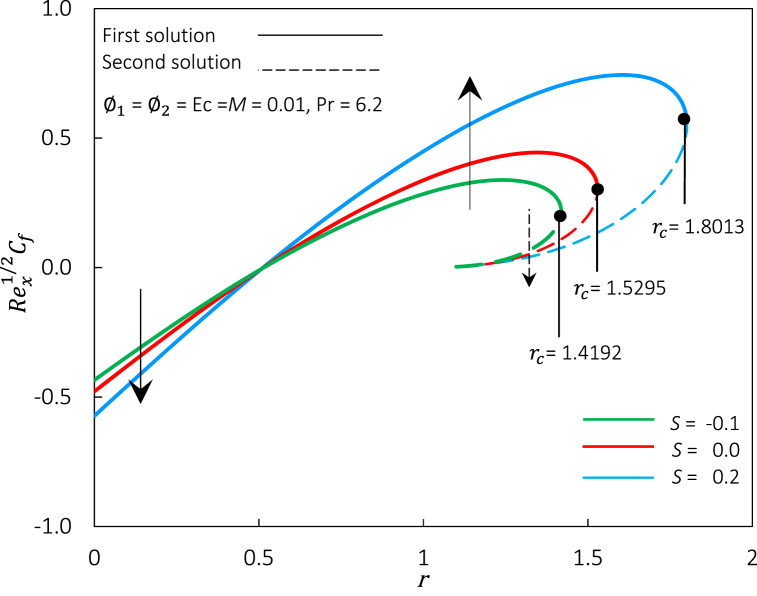
$Re_{x}^{1/2}C_{f}$ against r for various S.

**Fig. 3:**
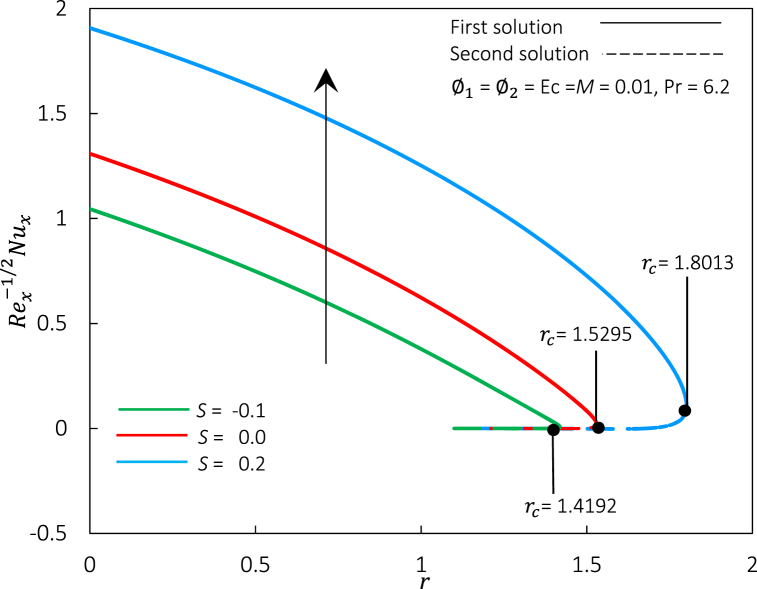
$Re_{x}^{-1/2}{Nu}_{x}$ against r for various S.

**Fig. 4:**
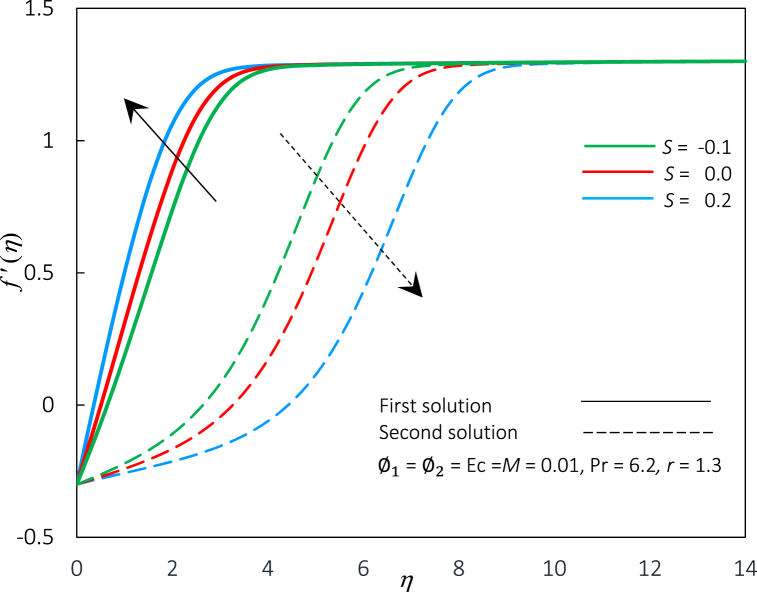
The impact of parameter S on velocity profiles $f^{\prime }\left(\eta \right)$.

**Fig. 5:**
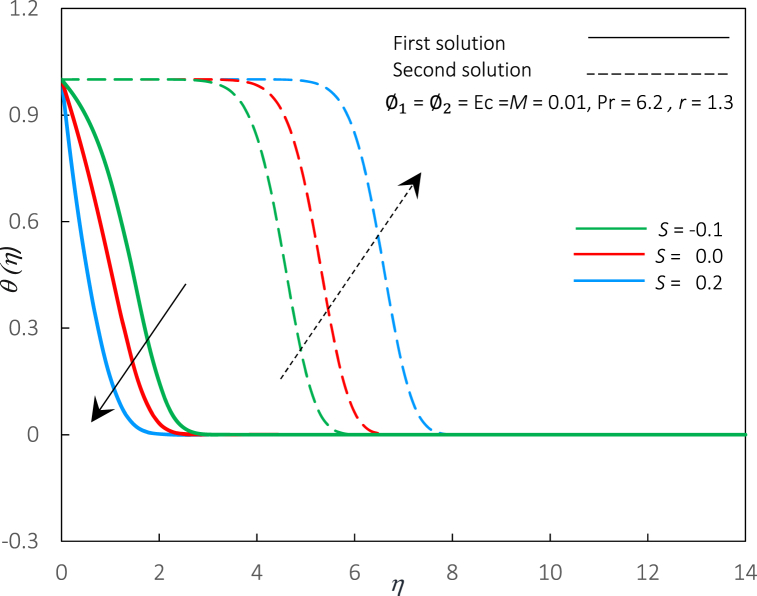
The impact of parameter S on temperature profiles θ(η).

**Fig. 6:**
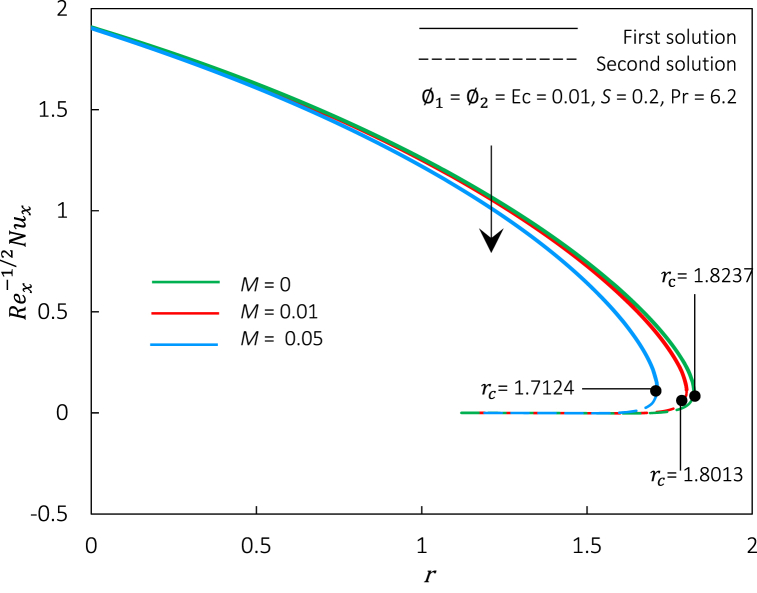
$Re_{x}^{1/2}C_{f}$ against r for various M.

**Fig. 7:**
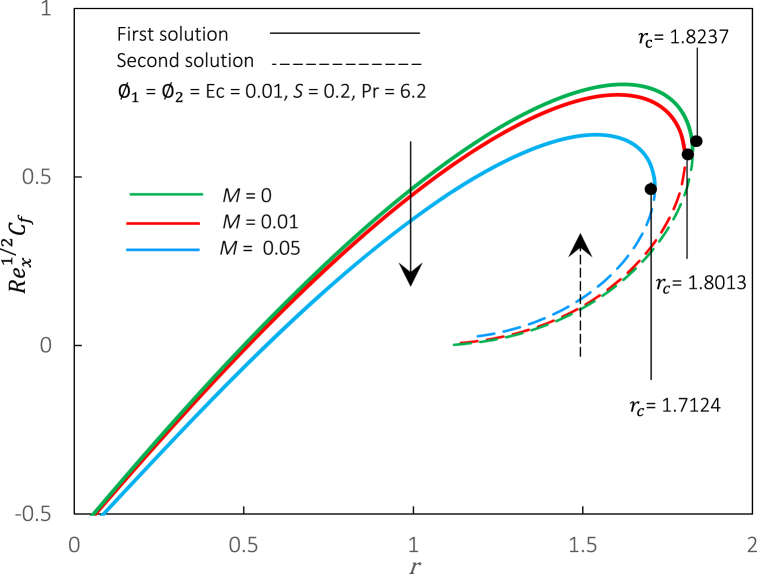
$Re_{x}^{-1/2}{Nu}_{x}$ against r for various M.

**Fig. 8:**
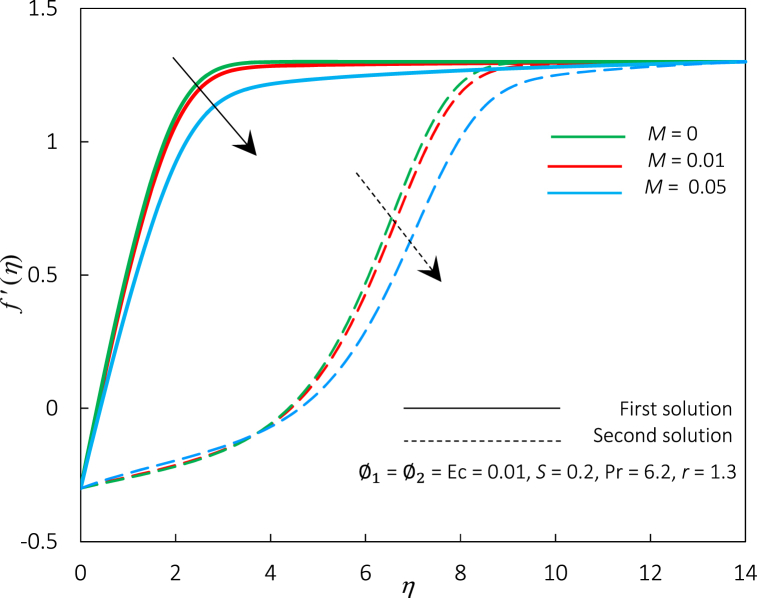
The impact of parameter *M* on velocity profiles $f^{\prime }\left(\eta \right)$.

**Fig. 9:**
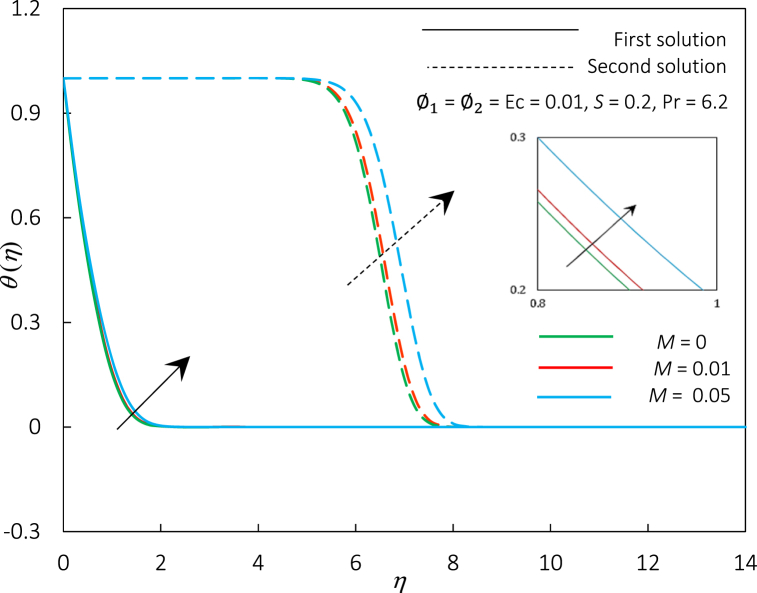
The impact of parameter *M* on temperature profiles θ(η).

**Fig. 10:**
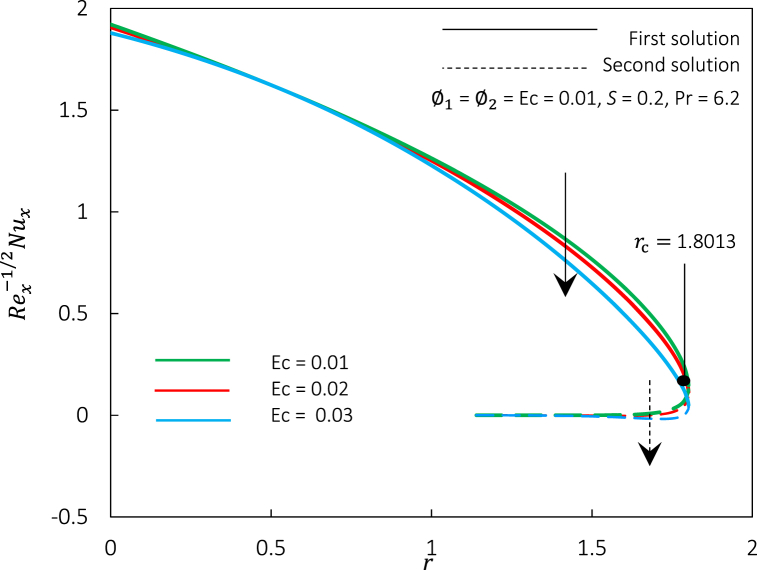
The impact of Ec number on velocity profiles $f^{\prime }\left(\eta \right)$.

**Fig. 11:**
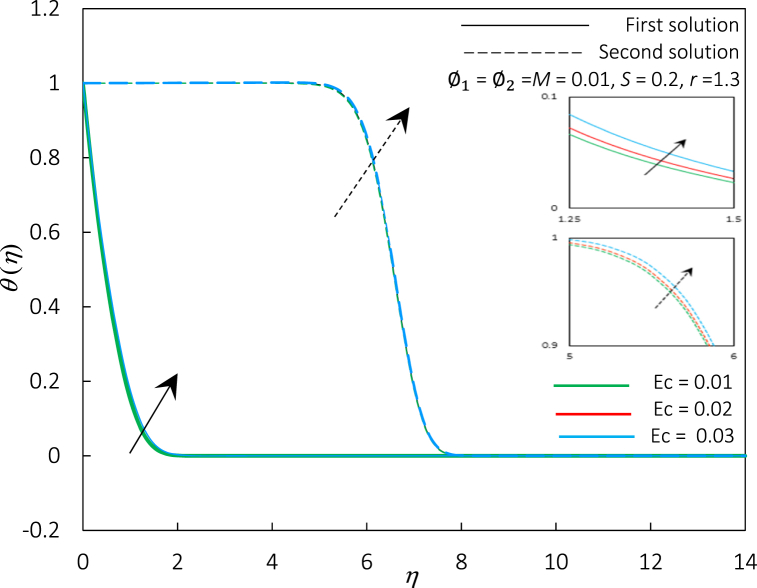
The impact of Ec number on temperature profiles θ(η).

**Fig. 12:**
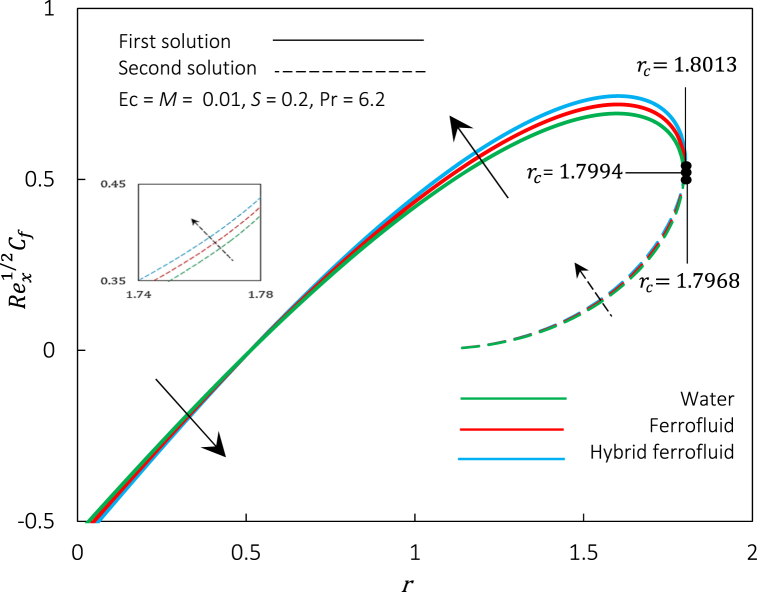
$Re_{x}^{1/2}C_{f}$ against r for various fluid.

**Fig. 13:**
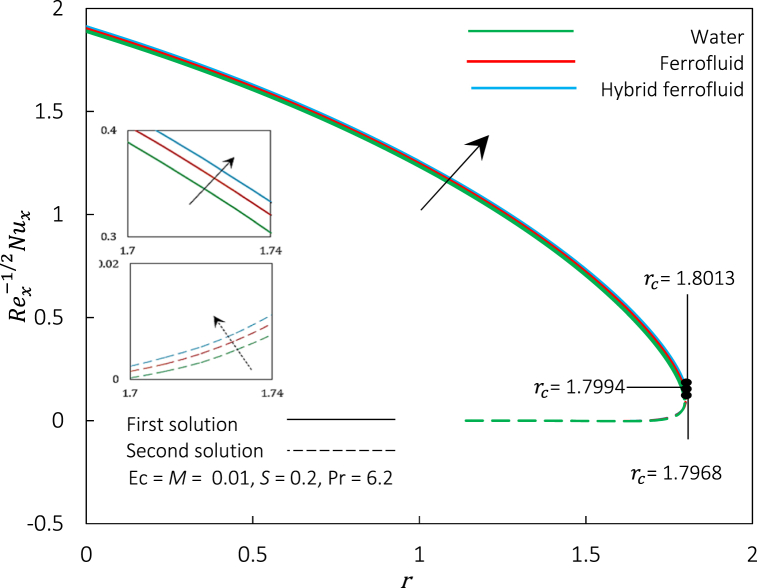
$Re_{x}^{-1/2}{Nu}_{x}$ against r for various fluid.

**Fig. 14:**
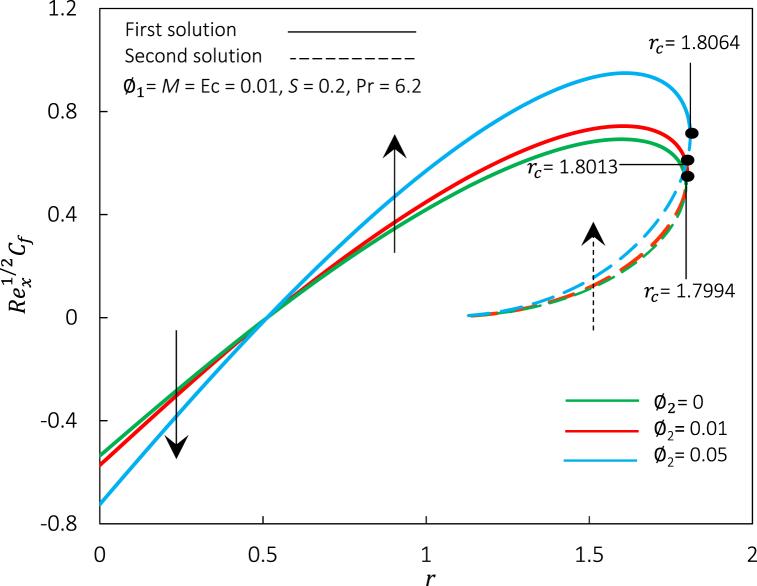
$Re_{x}^{1/2}C_{f}$ against r for various $\varphi_{2}$.

**Fig. 15:**
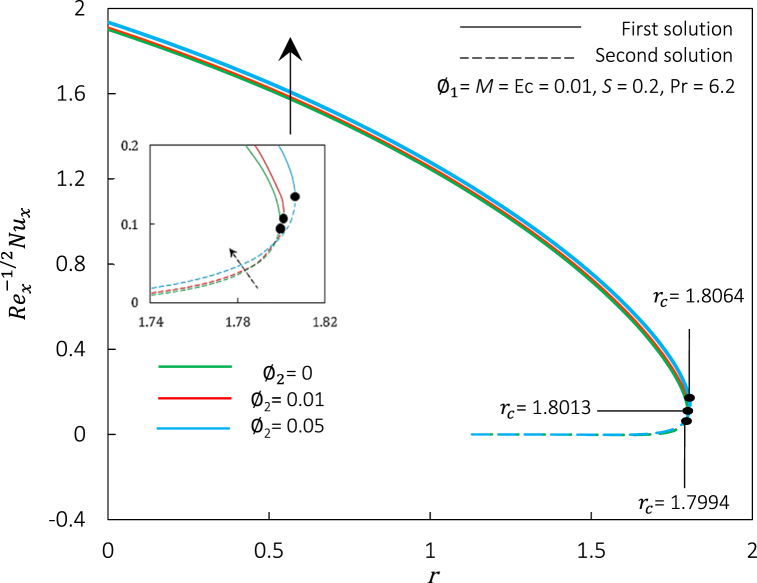
$Re_{x}^{-1/2}{Nu}_{x}$ against r for various $\varphi_{2}$.

**Fig. 16:**
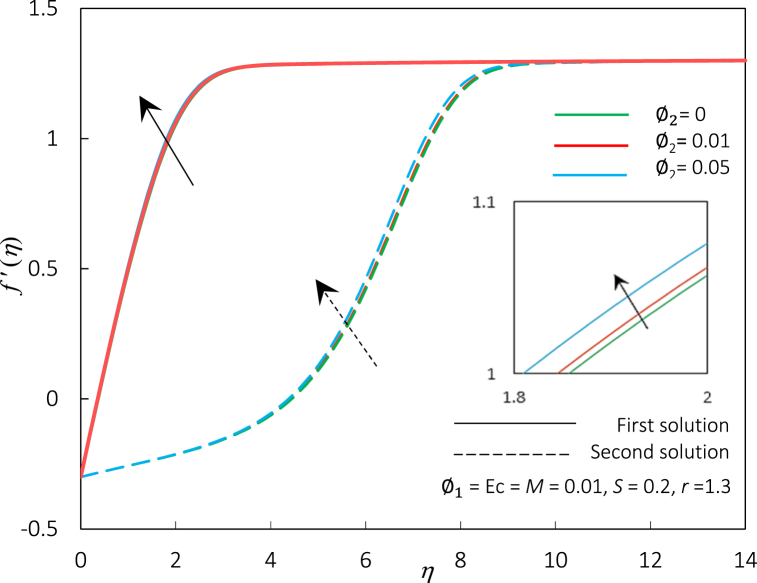
The impact of $\varphi_{2}$ on temperature profiles $f^{\prime }\left(\eta \right)$.

**Fig. 17:**
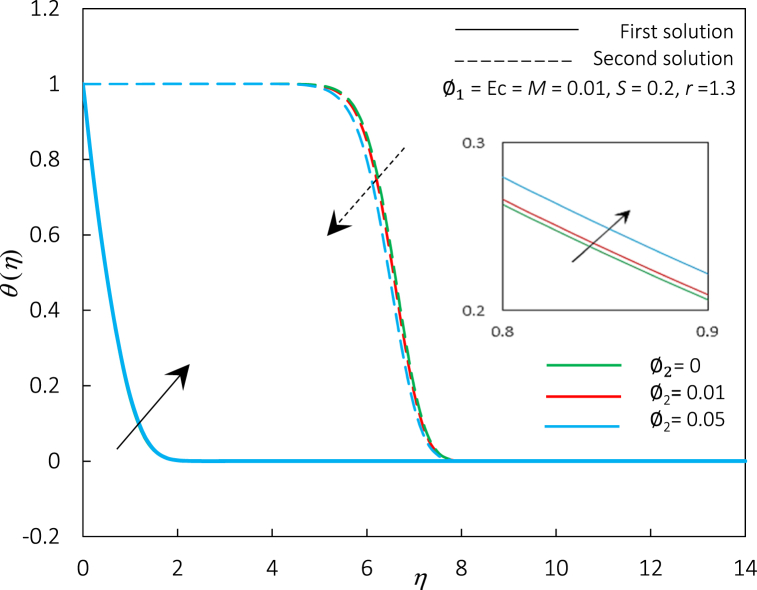
The impact of $\varphi_{2}$ on temperature profiles θ(η).

**Fig. 18:**
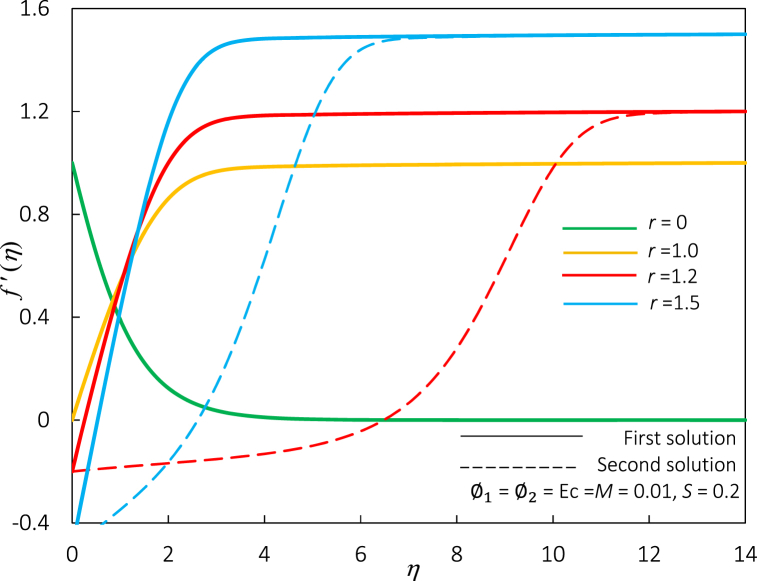
The impact of r on velocity profiles $f^{\prime }\left(\eta \right)$.

**Fig. 19:**
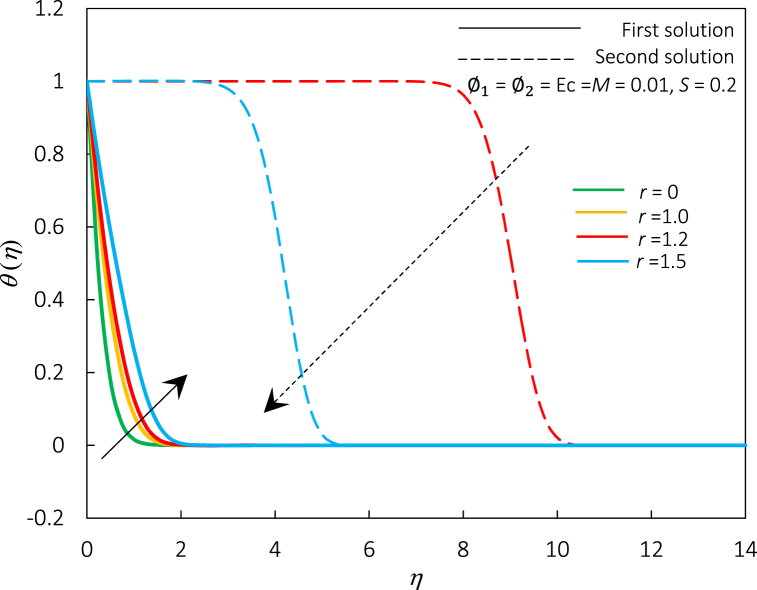
The impact of r on temperature profiles θ(η).

In this present work, the influence of various governing parameters, such as nanoparticle volume fraction $\varphi_{1}$ and $\varphi_{2}$, magnetic parameter *M*, Eckert number Ec, suction *S,* and moving parameter *r* are considered in the problem. During numerical computation in MATLAB, values $\varphi_{1}$ = 0.01, $\varphi_{2}$ = 0.01, *M* = 0.01, Ec = 0.01, and *S* = 0.2 are fixed and can be changed based on various governing parameters selected. Best to remark, the local skin friction coefficient $Re_{x}^{1/2}C_{f}$ is a dimensionless quantity often used to characterize the level of skin friction on a surface. It is defined as the ratio of the local wall shear stress to the dynamic pressure of the fluid. Meanwhile, the local Nusselt number $Re_{x}^{-1/2}{Nu}_{x}$ is a dimensionless quantity defined as the ratio of convective to conductive heat transfer across the boundary layer at the solid-fluid interface. Thus, this section reveals the significant impact of pertinent governing parameters towards the practical interest of flow behavior and heat transfer characteristics, along with a stability analysis for the duality of solutions. The finding obtained is further discussed in Fig. 2, Fig. 3, Fig. 4, Fig. 5, Fig. 6, Fig. 7, Fig. 8, Fig. 9, Fig. 10, Fig. 11, Fig. 12, Fig. 13, Fig. 14, Fig. 15, Fig. 16, Fig. 17, Fig. 18, Fig. 19 and Table 5.

**Table 5:** The smallest eigenvalues for variations of M and r when $\varphi_{1}=\varphi_{2}=Ec=0.01$ and S=0.2..

M	r	$\gamma_{1}$ (First solution)	$\gamma_{1}$ (Second solution)
0	1.6	0.6420	−0.2376
	1.7	0.4856	−0.1966
	1.8	0.2423	−0.0683
0.01	1.6	0.5975	−0.2229
	1.7	0.4377	−0.1743
	1.8	0.1089	−0.0274
0.05	1.5	0.5576	−0.1843
	1.6	0.4147	−0.1569
	1.7	0.1718	−0.0284

Fig. 2 presents the variations of $Re_{x}^{1/2}C_{f}$ versus r for S=−0.1,0,and0.2. The value of $Re_{x}^{1/2}C_{f}$ is zero at r=1/2, negative when r<1/2, and positive when r>1/2. Physically, the non-negative $Re_{x}^{1/2}C_{f}$ indicates that the fluid strives a resistive force on the sheet, while a negative value indicates the contrary. $Re_{x}^{1/2}C_{f}$ does not occur at r=1/2, demonstrating that the velocity of the sheet and the fluid are identical. Note that two solutions are found at r>1, and a unique solution is obtained at r≤1. However, no solution can be discovered within the vicinity of $r>r_{c}$, where $r_{c}$ is the critical/turning point of r because of the boundary layer's separation from the surface. Raised the *S* parameter value implies an increment in critical value where $r_{c}=1.4192,1.5295,and\;1.8013\text{,}$ indicating that the suction impact contributes to the postponement of the boundary layer separation process. The event's physical mechanism is due to the permeable sheet aiding in maintaining laminar flow by entrapping molecules that move slowly along the moving surface. Thus, the wall shear stress at the sheet's surface rises, delaying the flow separation. The first solution in Fig. 2 shows that when r<0.5, an enlargement of S improves the sheet's permeability, contributing to the capture of low-speed molecules and thus increasing the $Re_{x}^{1/2}C_{f}$, however, a contrary result was obtained by the second solution. The second solution relates to bifurcations in the solutions. The nonlinearity of the ordinary differential equations and variations in the moving parameter or other governing parameter mostly generates it. The lessening values of $Re_{x}^{1/2}C_{f}$ signify the decrement of the frictional drag imparted into the moving surface, which is beneficial in maintaining the laminar boundary layer.

Further, as illustrated in Fig. 3, the $Re_{x}^{-1/2}{Nu}_{x}$ increased proportionally to an increment of S. Additionally, even $Re_{x}^{1/2}C_{f}$ zero at r=1/2 (refer Fig. 2), but the sheet transfers its heat to the fluid for a positive $Re_{x}^{-1/2}{Nu}_{x}$ due to temperature differences. Physically, as values of *S* grow, the sheet's permeability increases, allowing more hybrid ferrofluid to permeate through it. The excellent thermal conductivity of hybrid ferrofluid boosts the heat flux at the moving sheet. Suction has a faster heat transfer rate than an injection, decelerating the separation of the boundary layer, lowering the thermal boundary layer's density, and increasing the heat transfer rate with the gradient's temperature. Thereby increasing flow close to the wall as S grows.

The profiles (velocity and temperature) for distinctive S values are shown in Fig. 4, Fig. 5. Each profile exhibits that the boundary conditions are met, supporting the accuracy of the solutions obtained. From Fig. 4, as the value of S is enlarged, the first solution's velocity profiles rise dramatically, whereas the second solution decreases significantly. Subsequently, the momentum boundary layer thickness is reduced in the first solution and increases in the second solution. Practically, through the permeable walls, suction augments the mass withdrawn from the laminar boundary layer, resulting in the fluid migrating to unoccupied regions and affecting the surface limits. Thus, the shear stress at the wall will grow as the fluid flow velocity increases, and this finding is consistent with Fig. 1. In contrast, for temperature profiles (refer to Fig. 5), the increments of S reduce the temperature profiles and the thickness of thermal boundary layer in the first solution. However, a contrary remark is made regarding an alternative solution. Practically, reducing thermal boundary layer thickness contributes to increased heat flux production. As a result of this process, heat transfer performance improves. This finding is similar to the observation in Fig. 2, where the $Re_{x}^{-1/2}{Nu}_{x}$ rises with an improvement in S.

The magnetic parameter M, representing the MHD effect, has been incorporated into the momentum Eq. (8). Thus, an impact of M on $Re_{x}^{1/2}C_{f}$ and $Re_{x}^{-1/2}{Nu}_{x}$ in hybrid ferrofluid flow can be illustrated in Fig. 6, Fig. 7. We perceived that $Re_{x}^{1/2}C_{f}$ and $Re_{x}^{-1/2}{Nu}_{x}$ decreased when M is upsurged in the hybrid ferrofluid flow past a permeable surface. The decrement of $Re_{x}^{1/2}C_{f}$ and $Re_{x}^{-1/2}{Nu}_{x}$ shows that the movement of fluid velocity is slower than before, and the temperature of heat being transferred to the fluid also slows down. The physical explanation for this event may be due to suppressed turbulence, which decreased the velocity profiles. The formation of the Hartmann layer (thermal insulating layer) near the surface reduces the heat transfer rate as the magnetic field strength intensifies in a conducting fluid. Meanwhile, the critical values for an increment of M=0,0.01,0.05 are $r_{c}=1.8237\text{,}$ 1.8013,and1.7124, respectively, which indicates that the magnetic parameter postponed the boundary layer separation and appears to reduce the solution's range. Physically, an increment of the magnetic parameter in hybrid ferrofluid flow can be attributed to the suppression of turbulence and alteration of the boundary layer profile due to the interaction between the magnetic field and the suspended particles. Thus, the precise cause of the delay in boundary layer separation may vary on the geometry and circumstances of the problem. Generally, it is connected to the magnetic field's capacity to stabilize the boundary layer and govern the fluid dynamics.

Fig. 8, Fig. 9 portray the behaviour of the profiles for temperature and velocity for an accumulating amount of M’s value. The velocity profiles significantly decrease while temperature profiles are increased, which causes the thickness of the thermal and momentum boundary layer for the first and second solutions to increase. It is also apparent from these graphs that the domain of dual solution occurrence is narrow as the magnetic parameter is up-surge. Physically, the rise of M value in the flow creates and increases the resistive type of force called Lorentz force; a phenomenon occurs when a magnetic field inhibits fluid mobility perpendicular to the field lines, but it facilitates fluid motion along the field lines. Essentially, it will generate substantially more excellent flow resistance and elevates temperature. Thus, the fluid velocity profiles become more uniform in the direction of the magnetic field and less uniform in the perpendicular direction, resulting in a drop in the velocity profile. The Lorentz force also produces heat-generating electrical currents in the fluid, causing an increment in the fluid's temperature profile. The magnetic field may also modify the thickness of the thermal and momentum boundary layers, which are portions of a fluid close to a solid surface with a temperature and velocity gradient, respectively.

The Eckert number Ec, has been integrated into the energy Eq. (9) to measure the viscous dissipation effects. Hence, Fig. 10, Fig. 11 depict the influence of Ec on the $Re_{x}^{-1/2}{Nu}_{x}$ and θ(η), respectively. Fig. 10 shows that $Re_{x}^{-1/2}{Nu}_{x}$ of hybrid ferrofluid was decreased, but temperature profiles in Fig. 11 grew due to a rise in Ec for the first and second solutions. It was also noticeable that the thermal boundary layer thickness was upraised, indicating that the temperature of the hybrid ferrofluid could be adjusted by varying the viscous dissipation effects. The Ec number increases fluid kinetic energy, which increases viscous dissipation heat. Due to fluid-surface friction, viscous dissipation converts kinetic energy into heat energy. Thus, the $Re_{x}^{-1/2}{Nu}_{x}$, which measures surface convective heat transport, decreases as temperature rises. Additionally, as the Eckert number rises, the fluid's kinetic energy outweighs its thermal energy, increasing momentum near the solid's surface. This momentum enhances surface mixing and turbulent flow, thus thickening the thermal boundary layer. According to Mabood et al. [72], an increment of viscous dissipation effects permitted the energy to be kept inside the proximity of fluid. Dissipation is caused by viscous and elastic distortion, which generates heat due to energy dissipation (frictional heating). By the agreement, the effects of viscous dissipation can be utilized as a regulating element in conducting flows to monitor the percentage of hybrid ferrofluid’ cooling. In this study, the viscous dissipation impact in hybrid ferrofluid is unable to control the thermal boundary layer separation as shown in Fig. 10. It is due to the dominance of magnetic strength and particle concentration in the fluid and the non-linear relationship between fluid velocity and skin friction.

Fig. 12, Fig. 13 exemplify the graphical representation of $Re_{x}^{1/2}C_{f}$ and skin friction coefficient, respectively, for dissimilar nanoparticles' volume fraction $\varphi_{1}$ and $\varphi_{2}$ representing different fluids to inspect the heat transfer performance of hybrid ferrofluid against ferrofluid and water. Both figures illustrate three sets of nanoparticles volume fraction where; set (a): $\varphi_{1}=\varphi_{2}=0$ indicate water, set (b): $\varphi_{1}=0.01,\varphi_{2}=0$ indicates ferrofluid, and set (c): $\varphi_{1}=\varphi_{2}=0.01$ indicates hybrid ferrofluid. Through an increment value of $Re_{x}^{1/2}C_{f}$ and $Re_{x}^{-1/2}{Nu}_{x}$ from set (a) to set (c), both figures show that the hybrid ferrofluid's frictional drag and heat transfer rate is the best, followed by ferrofluid and water. Physically, combining the magnetic and thermal properties of two ferroparticles can lead to better control over the fluid flow, higher thermal conductivity, and altered interfacial behaviour at the fluid-solid interface. Additionally, hybrid ferrofluid can be more stable than ferrofluid and water due to better dispersion and stabilization of the ferroparticles hybridization. The changes from a regular fluid to a hybrid ferrofluid by the enhancement of volume concentration of $CoFe_{2}O_{4}$, accelerating the boundary layer separation. The physical mechanism behind the event is due to the magnetic interactions between the ferroparticles, which form thicker and stronger magnetic boundary layers near solid surfaces. Thus, this study reveals that hybrid ferrofluid's cooling is significantly faster than in ferrofluid and water, which may take a while. This finding is in line with the study by Ref. [37].

Fig. 14, Fig. 15 represent the variations of $Re_{x}^{1/2}C_{f}$ and $Re_{x}^{-1/2}{Nu}_{x}$, accordingly, for different values of $\varphi_{2}$. Fig. 14, Fig. 15 express an augmentation in $\varphi_{2}$ automatically increased the shear stress and thermal conductivity, respectively. Physically, the increase in $Re_{x}^{1/2}C_{f}$ is due to the rise in ferroparticle colloidal suspension, which enhances the collision of ferrofluid nanoparticle dispersion. Thus, increasing the nanoparticles' concentration ratio may also boost their synergistic impact, improving the heat transfer rate. Additionally, the rise of $\varphi_{2}$ escalates the hybrid ferrofluid viscosity and subsequently boosts the fluid velocity, as shown in Fig. 16. The velocity profiles also show that the momentum boundary layer thickness is dropped in agreement with the increment of $\varphi_{2}$ for the first and second solutions. The temperature profiles in Fig. 17 align with the ornament, as indicated in Fig. 15. The rise in hybrid ferrofluid temperature enhances the hybrid ferrofluid heat conductivity and gradually lowers the rate of thermal convection. The thickness of the thermal boundary layer grows for the first solution while shrinking in the second solution.

Fig. 18, Fig. 19 show profiles (velocity and temperature) for different values of r. The raised value of r leads to an enhancement of the temperature and velocity profiles of the first solution. In Fig. 18, the thickness of the momentum boundary layer is diminished for the first and second solutions. The thermal boundary layer thickness is elevated for the first solution but degrades for the other solution (refer to Fig. 19). Recall that a dual solution occurs for r>1. Thus, no second solution appears for r=0 and r=1 in both figures.

The linearized Eqs. (23), (24) with boundary conditions (26) are solved using a bvp4c MATLAB to assess the reliability of the solutions. The sign of the smallest eigenvalues is crucial to verify the solutions' stability. Positive eigenvalues denote a stable solution, which physically implies a modest disturbance and is not disruptive to the flow. In contrast, eigenvalues with negative signs indicate an unstable solution, which explains the rise in disturbances. Thus, Table 5 reveals that the second solution is unstable, whereas the first solution is stable.

## 6 Conclusion

The problem of hybrid ferrofluid flow and heat transfer over a moving permeable surface in a parallel stream with MHD, viscous dissipation, and suction had been solved numerically. These final discoveries focused on the specified model configuration for various parameter values however limited to a single-phase Tiwari and Das model for a hybrid ferrofluid flow. Thus, hybridizing nanoparticles other than magnetic nanoparticles may provide different results. This work should be extended using the Buongiorno model to evaluate Brownian motion and thermophoresis effects. Thermal radiation, Joule heating, mixed convection, slip conditions, and complex geometries should be considered to improve current findings. The other impacts and sophisticated geometry involved could provide a deeper grasp of the actual situation and bring new insight into the subject, particularly for those engaged in the hybrid nanofluid domain. Thus, the highlighted outcomes of this study can be concluded as follows:

• Hybrid ferrofluid possesses better convective heat transfer and skin friction than mono-ferrofluid and water.
• A dual solution is obtained at r>1. The stability analysis confirmed that the first solution is stable and physically realizable, whereas the second is unstable.
• Heat transfer rate, skin friction coefficient, and velocity profile are enhanced by boosting *S* and $\varphi_{2}$ while oppositely for *M.*
• The temperature profile is increased with the increment of $\varphi_{2}$, *M,* and Ec number but declined by *S.*
• Ec has deteriorated the heat transfer rate, surprisingly insignificantly influencing skin friction and boundary layer separation.
• The development of boundary layer separation is delayed by the increment of *S* and $\varphi_{2}$.

This research approach may benefit biomedical and industrial uses. A specific example of an application that may be adapted from this study approach is the development of microfluidic devices for controlled medication release. Hybrid ferrofluid flow may adequately manage the movement of medicine within a permeable surface under an external magnetic field. Suction can govern medicine release, while viscous dissipation regulates flow and reduces mixing. MHD effects allow ferrofluid magnetic and electrical properties to be studied, which improves medication delivery. Due to this combination of events, hybrid ferrofluid flow can improve drug delivery systems. Further applications include fluid mixing, thermal transfer augmentation, oil recovery, and environmental cleansing. Based on the findings of this study, the cooling/heating industries could improve their heat transfer operation by including a small-scale magnetic field and suction effects.

## Author contribution statement

Sakinah Idris: Performed the experiments; Analyzed and interpreted the data; Wrote the paper.

Anuar Jamaludin: Contributed reagents, materials, analysis tools or data; Wrote the paper.

Roslinda Nazar; Ioan Pop: Conceived and designed the experiments.

## Data availability statement

Data included in article/supp. material/referenced in article.

## Declaration of competing interest

The authors declare that they have no known competing financial interests or personal relationships that could have appeared to influence the work reported in this paper.
