# Expression of concern: “Heat transfer characteristics of magnetized hybrid ferrofluid flow over a permeable moving surface with viscous dissipation effect” [Heliyon 9 (2023) e15907]

**DOI:** 10.1016/j.heliyon.2025.e43818

**Published:** 2025-09-06

An investigation conducted on behalf of the journal by Elsevier's Research Integrity & Publishing Ethics team found a significant increase of citations to papers published by the authors, Ioan Pop and Roslinda Nazar, between the original submission and the revised version of this article. In summary, 16 papers by the authors were cited in the original version of the article. This increased to 23 papers in the revised version of the article. The Editors have determined that the references in the paper do not provide readers with a balanced reflection of the literature. Readers should take this into consideration when evaluating this paper.

